# Exploring the Potential of Flubendazole in Filariasis Control: Evaluation of the Systemic Exposure for Different Pharmaceutical Preparations

**DOI:** 10.1371/journal.pntd.0002838

**Published:** 2014-05-29

**Authors:** Laura Ceballos, Charles Mackenzie, Timothy Geary, Luis Alvarez, Carlos Lanusse

**Affiliations:** 1 Laboratorio de Farmacología, Centro de Investigación Veterinaria de Tandil (CIVETAN), CONICET, Facultad de Ciencias Veterinarias, UNCPBA, Tandil, Argentina; 2 Department of Pathobiology and Diagnostic Investigation, School of Veterinary Medicine, Michigan State University, East Lansing, Michigan, United States of America; 3 Institute of Parasitology, McGill University, Ste-Anne-de-Bellevue, Quebec, Canada; Centers for Disease Control and Prevention, United States of America

## Abstract

The goal of elimination of the human filariases would benefit greatly from the use of a macrofilaricidal agent. *In vivo* trials in humans and many experimental animal models suggest that flubendazole (FLBZ) is a highly efficacious macrofilaricide. However, since serious injection site reactions were reported in humans after parenteral FLBZ administration, the search for alternative pharmaceutical strategies to improve the systemic availability of FLBZ and its metabolites has acquired urgency in both human and veterinary medicine. The goal of the current work was to compare the systemic exposure of FLBZ formulated as either an aqueous hydroxypropyl-β-cyclodextrin (CD) or aqueous carboxymethyl cellulose (CMC) suspension or a Tween 80-based formulation (TWEEN) in rats and jirds (*Meriones unguiculatus*). Healthy animals of both species were allocated into four experimental groups of 44 animals each: FLBZ-CD_oral_ and FLBZ-CD_sc_, treated with the FLBZ-CD formulation by the oral or subcutaneous routes, respectively; FLBZ-TWEEN_sc_, dosed subcutaneously with the FLBZ-TWEEN formulation; and FLBZ-CMC_oral_, treated orally with the FLBZ suspension. The FLBZ dose was 5 mg/kg. FLBZ and its hydrolyzed (H-FLBZ) and reduced (R-FLBZ) metabolites were recovered in plasma samples collected from rats and jirds treated with the different FLBZ formulations. In both species, FLBZ parent drug was the main analyte recovered in the bloodstream. In rats, FLBZ systemic exposure (AUC_0-LOQ_) was significantly (P<0.05) higher after the FLBZ-CD treatments, both oral (4.8±0.9 µg.h/mL) and subcutaneous (7.3±0.6 µg.h/mL), compared to that observed after oral administration of FLBZ-CMC suspension (0.93±0.2 µg.h/mL). The same differences were observed in jirds. In both species, parenteral administration of FLBZ-TWEEN did not improve the systemic availability of FLBZ compared to FLBZ-CD_oral_ treatment. In conclusion, formulation approaches that enhance the availability of flubendazole in the rat and jird may have therapeutic implications for a drug with poor or erratic bioavailability.

## Author Summary

Lymphatic filariasis and onchocerciasis are tropical parasitic diseases caused by filarial nematodes, which constitute a serious public health issue in tropical regions. Lymphatic filariasis causes debilitating lymphedema and hydrocele, resulting in temporary or permanent disability. Onchocerciasis (also known as river blindness) causes visual impairment and blindness, constituting one of the leading causes of blindness in the world. The control of human filarial infections currently depends on strategies predominantly focused at killing microfilariae (larval stage) by the use of ivermectin or diethylcarbamzine, usually in combination with albendazole. It is now generally recognized that the success of filariasis control programs in a reasonable time-frame requires the addition of a macrofilaricide (adult stage) compound. Although flubendazole has demonstrated macrofilaricidal activity *in vivo*, the approved formulations provide almost no oral bioavailability. The search for alternative pharmaceutical strategies to improve the systemic availability of flubendazole has acquired urgency in both human and veterinary medicine. Searching for improved flubendazole absorption, different flubendazole pharmaceutical preparations were assessed, both in rats and jirds, in the study described here. The work demonstrated that flubendazole pharmacokinetics could be markedly modified by changes in drug formulation. The resulting improved systemic exposure of flubendazole may have a significant impact on its macrofilaricidal efficacy.

## Introduction

Lymphatic filariasis and onchocerciasis are tropical parasitic diseases caused by filarial nematodes in the superfamily *Filarioidea*, also known as “filariae”. Filariasis constitutes a serious public health issue in tropical regions. Approximately 128 million individuals suffer from lymphatic filariasis (commonly known as elephantiasis), mainly in Africa and South-East Asia. The disease causes debilitating lymphedema and hydrocele, resulting in temporary or permanent disability, impairment of physical productivity, income loss and social stigma [1]. Onchocerciasis [also known as river blindness) afflicts approximately 26 million individuals in Africa, where an estimated 746,000 are visually impaired and 265,000 are blinded by the disease, constituting one of the leading causes of blindness in the world [1]–[2]. Lymphatic filariasis is caused by *Wuchereria bancrofti*, *Brugia malayi* and *Brugia timori*, while *Onchocerca volvulus* is the cause of river blindness. Infective larvae of filariae are transmitted by blood-feeding insects, developing into fertile adults several months after infection. Chronic, long-term infections occur through suppression of host immunity [2].

Current control programs rely on three drugs, which are safe and available through donation: diethylcarbamazine (DEC), ivermectin (IVM), and albendazole (ABZ). DEC kills larval stages in the host (microfilaria) and provides long-term sterility of adults, with limited adulticidal efficacy in the regimens employed. It is contraindicated in areas where onchocerciasis is endemic, due to potentially serious and unacceptable side effects affecting the eyes and the skin of infected persons [2] as well as in pregnancy [3]. IVM is a microfilaricide and also provides long-term sterilization of adult worms, preventing re-population of the host with microfilariae for 6 months or longer, but needs to be given at least annually [4]. Like DEC, IVM has limited macrofilaricidal effects in humans or other animals, which greatly prolongs the time required for mass drug administration programs to progress to eradication [5]. Lastly, ABZ is routinely included with annual treatments of DEC or IVM in lymphatic filariasis control programs. Nevertheless, the activity of the benzimidazole component (ABZ) in this regimen is uncertain, and whether combination therapy confers benefits over DEC or IVM alone remains controversial [5]. Thus, the control of human filarial infections currently depends on strategies predominantly focused on killing microfilariae and the long-term cessation of their production [6]. It is now generally recognized that the success of filariasis control programs in a reasonable time-frame would be favored by the addition of a macrofilaricidal compound to current control strategies [4], [6], [7].

Flubendazole (FLBZ), a methylcarbamate benzimidazole (BZD), is highly active against a broad spectrum of gastrointestinal nematodes in humans and some animal species. FLBZ has also demonstrated a marked lethal effect on many filarial species in animal model hosts [6]. FLBZ has been reported to be the best macrofilaricidal molecule within the BZD group [8]. This compound is already approved for use in humans [9], which may be an advantage over other candidates for filariasis. FLBZ, like other BZDs, has limited water solubility and is commercially available for oral administration in humans as tablets or suspensions, providing low systemic bioavailability [10]. The macrofilaricidal activity of FLBZ is thought to require sustained systemic exposure, which is not achieved after administration of conventional oral formulations. The *in vivo* activity of FLBZ against a variety of filariid species has been reported after its parenteral administration in animal and human trials [6], [11]–[12]. FLBZ was available as a sterile suspension for intramuscular treatment [13]; however, since serious injection site reactions were reported in humans after parenteral FLBZ administration [12], the search for alternative pharmaceutical strategies to improve systemic availability of FLBZ after oral dosing has acquired urgency in both human and veterinary medicine.

Several pharmacotechnical strategies have been explored to enhance BZD systemic bioavailability. Cyclodextrins (CD), cyclic oligosaccharides used to increase drug solubility, are well-known molecular hosts capable of including water-insoluble guest molecules via non-covalent interaction within a hydrophobic cavity [14]. Enhanced aqueous solubility and bioavailability of guest molecules is a common effect observed after drug formulation with CD [15]. We have previously reported that incorporation of FLBZ into a hydroxypropyl-β-cyclodextrin (CD) formulation significantly increased its water solubility [16] and systemic exposure in mice by more than 25-fold compared to the conventional FLBZ suspension [17]. The relative bioavailability of albendazole sulphoxide (ABZSO) in mice was also increased by formulation with a CD [18]. Similar findings have been reported in humans [19].

The goal of the current study was to compare the plasma pharmacokinetic behaviour and systemic exposure of FLBZ formulated as either an aqueous CD-based solution (FLBZ-CD), aqueous carboxymethylcellulose (CMC) suspension (FLBZ-CMC) or a Tween 80-based formulation (FLBZ-TWEEN) in non-infected jirds (*Meriones unguiculatus*) and rats.

## Methods

### Chemicals

Pure reference standards of FLBZ, reduced-FLBZ (R-FLBZ) and hydrolyzed-FLBZ (H-FLBZ) used to develop the analytical methodology were kindly provided by Janssen Animal Health (Beerse, Belgium). Oxibendazole (OBZ), used as internal standard, was obtained from Schering Plough (Kenilworth, NJ, USA). HPLC grade acetonitrile and methanol were from Sintorgan S.A. (Buenos Aires, Argentina) and J.T. Baker (New Jersey, USA), respectively. HPBCDs were from ISP Pharmaceuticals (Cavasol, Cavitron, New Jersey, USA). Low viscosity grade sodium CMC was purchased from Anedra (Buenos Aires, Argentina). Tween 80 was purchased from Biopack (Buenos Aires, Argentina).

### Preparation of FLBZ formulations

The FLBZ CD-based solution was prepared by dissolving FLBZ (0.1%) and CD (10%) in deionized water. The pH of the formulation was adjusted to 1.2 using hydrochloric acid (25 mM). The formulation was shaken until total dissolution of the drug and then was filtrated through a 0.45 µm filter (Whatman, NJ, USA). The final FLBZ concentration was confirmed by HPLC (n = 4). Cavitron and Cavasol were the CD used in formulations intended for oral and parenteral administration, respectively. The Tween 80-based formulation was prepared by dissolving FLBZ (0.25%) in Tween 80. The FLBZ-suspension was prepared by addition of FLBZ (0.1%) and CMC (0.1%) in deionized water (pH = 6.0) with shaking for 6 h. The FLBZ- CMC suspension was vigorously shaken immediately before intragastric administration to jirds and rats. FLBZ formulations were freshly prepared and maintained under refrigeration (3–5°C).

### Experimental animals

#### Ethics statement

One hundred and seventy six (176) healthy jirds (approx. 50 g) and the same number of Wistar rats (approx. 200 g) were housed in a controlled temperature (21±2°C), light-cycled (12 h light/dark cycle) room. Food and water were provided *ad libitum*. Animal procedures and management protocols were approved by the Ethics Committee according to the Animal Welfare Policy (act 087/02) of the Faculty of Veterinary Medicine, Universidad Nacional del Centro de la Provincia de Buenos Aires (UNCPBA), Tandil, Argentina (http://www.vet.unicen.edu.ar) and followed the Guide for the Care and Use of Laboratory Animals (National Research Council, Washington DC, National Academy Press, 2011) [20].

#### Experimental design

Jirds and rats were randomly allocated into four experimental groups (44 animals each). Experimental animals received the following treatments: FLBZ-CD_oral_, dosed orally (administered volume 0.25 and 1 mL for jirds and rats, respectively) in a CD-based solution (Cavasol); FLBZ-CD_sc_, dosed subcutaneously (sc) (injection volume of 0.25 and 1 mL for jirds and rats, respectively) in a CD-based solution (Cavitron); FLBZ-TWEEN, dosed sc (injection volume of 0.1 and 0.4 mL for jirds and rats, respectively) in a Tween-based formulation; and FLBZ-CMC, dosed orally (same volume as for FLBZ-CD_oral_) in a CMC-based suspension. The oral dose was administered using an intragastric tube. All treatments were given as a single dose of 5 mg/kg. The dose was selected because it is the dose previously reported in efficacy studies performed in the murine model [17]. At defined time-points following treatment (5, 15 and 30 min, 1, 2, 3, 4, 6, 9, 12 and 16 h), blood samples were collected (n = 4 per time point) in heparinized plastic tubes. Plasma was separated by centrifugation at 2000*× g* for 15 min, placed into plastic tubes and frozen at −20°C until analysis by HPLC to quantify FLBZ, R-FLBZ and H-FLBZ plasma concentrations. After sc administration of the formulations, potential injection site reactions were evaluated by direct visual observation at the sampling time.

### Analysis of FLBZ and its metabolites

Chromatography was performed on a Shimadzu HPLC platform (Shimadzu Corporation, Kyoto, Japan), with two LC-10AS solvent pumps, an automatic sample injector (SIL-10A) with a 50 µL loop, an ultraviolet-visible spectrophotometric detector (UV) (SPD-10A) reading at 292 nm, a column oven (Eppendorf TC-45, Eppendorf, Madison, WI, USA) set at 30°C, and a CBM-10A integrator. Data and chromatograms were collected and analyzed using the Class LC10 software (SPD-10A, Shimadzu Corporation, Kyoto, Japan). The C18 reversed-phase column (5 µm, 250 mm×4.6 mm) was Kromasil (Kromasil®, Sweden). Elution from the stationary phase was carried out at a flow rate of 1.2 mL/min using an acetonitrile (34%)/ammonium acetate buffer (0.025 M, pH 5.3, 66%) as a mobile phase.

Plasma samples (100 or 200 µL for jirds and rats, respectively) were spiked with OBZ as internal standard. After 5 min, plasma samples were mixed with water up to 1 mL and the analytes were extracted using disposable C18 cartridges (Strata, Phenomenex, CA, USA) as previously described [17]. Identification of FLBZ and its metabolites was undertaken by comparison with the retention times of pure reference standards. Complete validation of the analytical procedures for extraction and quantification of drug and metabolites in plasma was performed before starting the analysis of experimental samples. Retention times for H-FLBZ, R-FLBZ and FLBZ were 5.7, 7.1 and 14.4 min, respectively. The calibration curves for each analyte, constructed by least squares linear regression analysis, showed good linearity with correlation coefficients ≥0.998. The limit of quantification (FLBZ and metabolites was 0.01 µg/mL), defined as the lowest measured concentration with a CV <20%, accuracy of ±20% and absolute recovery ≥70%.

### Pharmacokinetic analysis of the data

The peak concentration (Cmax) and time to peak concentration (Tmax) were read from the plotted concentration–time curve for each analyte. The area under the concentration–time curve from 0 up to the limit of quantification (AUC_0-LOQ_) for FLBZ and metabolites was calculated by the trapezoidal rule [21], using the PKSolutionTM computer program (Summit Research Services, Ashland, OR, USA).

### Statistical analysis

PK parameters are presented as arithmetic means ± SD. Non-parametric (Mann-Whitney) tests were used for statistical comparison of the pharmacokinetic data obtained from the experimental groups in each animal species. A value of P<0.05 was considered statistically significant. Statistical analysis was performed using the Instat 3.0 Software (Graph Pad Software, CA, USA).

## Results

**Figure 1:**
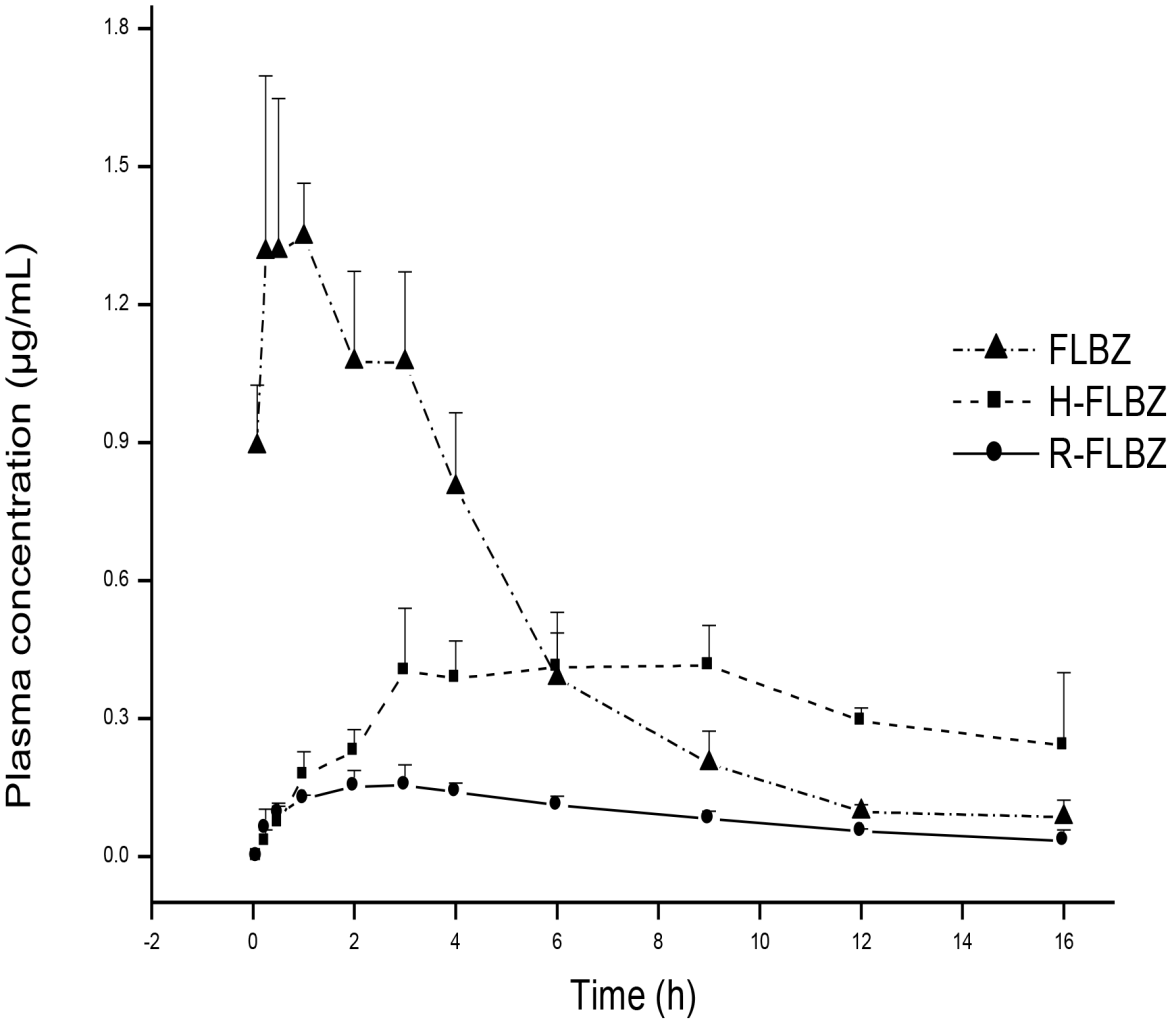
Rat. Mean (± SD) plasma concentrations of flubendazole (FLBZ) and its reduced (R-FLBZ) and hydrolyzed (H-FLBZ) metabolites following subcutaneous (sc) administration of FLBZ-CD solution (5 mg/kg) to uninfected rats (n = 4 per time point).

**Figure 2:**
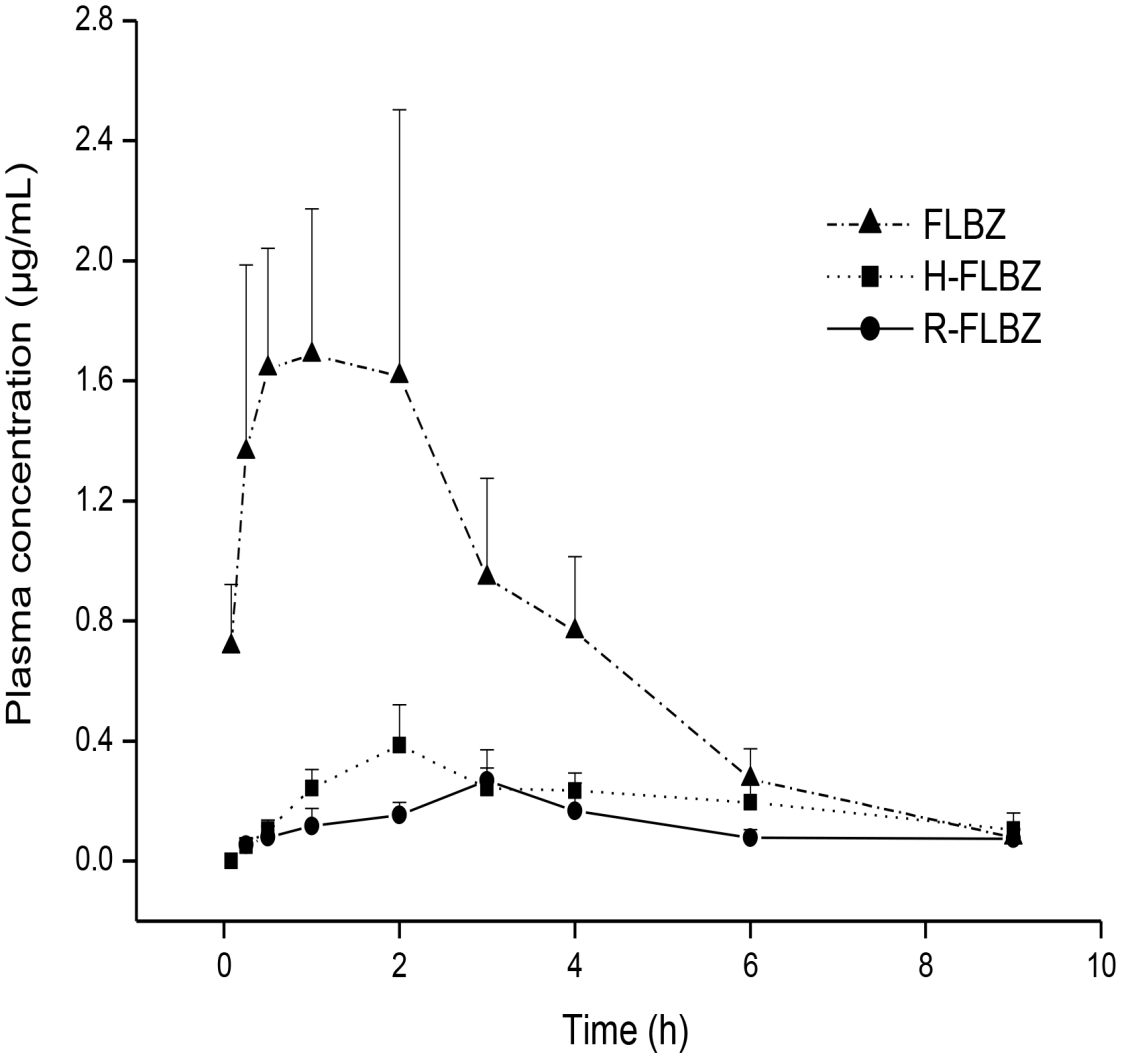
Jird. Mean (± SD) plasma concentrations of flubendazole (FLBZ) and its reduced (R-FLBZ) and hydrolyzed (H-FLBZ) metabolites following subcutaneous (sc) administration of FLBZ-CD solution (5 mg/kg) to uninfected jirds (n = 4 per time point).

No local tissue effects were observed in either species after sc administration of FLBZ formulated with either CD- or Tween 80. Figures 1 and 2 show mean plasma concentrations of FLBZ and metabolites after sc administration (5 mg/kg) of FLBZ in a CD-based formulation (FLBZ-CD_sc_) to rats and jirds, respectively. FLBZ and H-FLBZ were the main molecules detected in plasma of FLBZ-treated rats and jirds. Low R-FLBZ concentrations were detected between 15 min and 12 h post-treatment, with AUC_0-LOQ_ values about 10% of the total drug recovered from plasma in the different groups for both species. FLBZ and H-FLBZ concentrations rapidly increased to reach peak plasma concentrations, observed as early as 0.7–3.2 h (FLBZ) and 3.0–5.2 h (H-FLBZ), according the experimental group and animal species.

**Figure 3:**
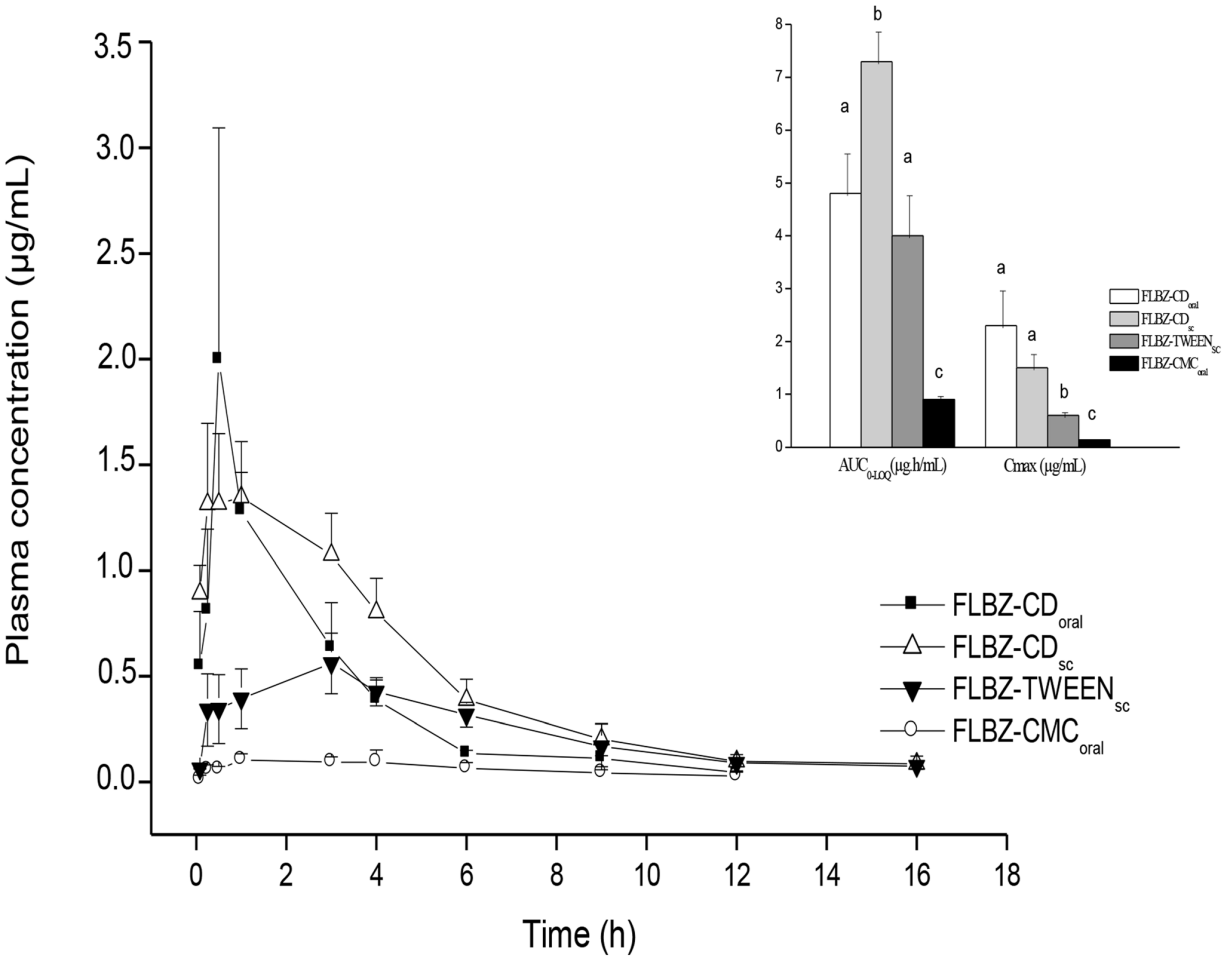
Flubendazole rat. Comparative flubendazole (FLBZ) mean (± SD) plasma concentration profiles obtained after administration of different formulations to uninfected rats (n = 4 per time point). Insert shows the pharmacokinetic parameters AUC _0-t_ and Cmax (µg/mL) estimated for FLBZ. Different letters indicate statistically significant differences (P<0.05) between experimental groups.

The comparative plasma concentration profiles of FLBZ obtained after oral or sc administration as different formulations to rats, along with some pharmacokinetic parameters (Cmax and AUC_0–LOQ_), are shown in Figure 3. Table 1 summarises the plasma pharmacokinetics parameters (Cmax, Tmax and AUC_0-LOQ_) for FLBZ and H-FLBZ obtained after oral or sc administration of the FLBZ formulations to rats. Higher drug systemic exposure was obtained after administration of FLBZ as a CD or Tween 80-based formulation to rats compared to the CMC-based suspension, resulting in significantly higher Cmax and AUC_0–LOQ_ values for both FLBZ H-FLBZ in the FLBZ-CD_oral_, FLBZ-CD_sc_ and FLBZ-TWEEN groups (Table 1).

**Table 1:** Pharmacokinetic parameters (mean ± SD) obtained for flubendazole (FLBZ) and its hydrolyzed metabolite (H-FLBZ) after treatment (5 mg/kg) with different FLBZ formulations to uninfected rats: Cyclodextrin-based (FLBZ-CD_oral_ or FLBZ-CD_sc_), Tween-based (FLBZ-TWEEN) and carboxy methylcellulose-based (FLBZ-CMC).

Pharmacokinetic parameters	FLBZ			
	FLBZ-CD_oral_	FLBZ-CD_sc_	FLBZ-TWEEN_sc_	FLBZ-CMC_sc_
**Cmax (µg/mL)**	2.34±0.75^a^	1.51±0.29^a^	0.60±0.07^b^	0.14±0.01^c^
**Tmax (h)**	0.87±0.75^a^	0.70±0.35^a^	2.50±1.00^a^	3.25±1.50^a^
**AUC_0-LOQ_ (µg.h/mL)**	4.80±0.86^a^	7.32±0.59^b^	4.00±0.80^a^	0.93±0.16^c^

	H-FLBZ			
**Cmax (µg/mL)**	0.72±0.39^a^	0.50±0.06^a^	0.56±0.07^a^	0.12±0.01^b^
**Tmax (h)**	3.25±0.50^a^	5.25±2.87^a^	5.00±1.15^a^	3.00±1.41^a^
**AUC_0-LOQ_ (µg.h/mL)**	4.37±1.37^a^	5.15±0.75^a^	5.32±0.41^a^	0.51±0.27^c^

C_max_: peak plasma concentration; T_max_: time to peak plasma concentration; AUC_0**-LOQ**_: area under the concentration vs. time curve from 0 up to the limit of quantification. *Different letters indicate statistically significant differences (P<0.05) between experimental groups*.

The sc administration of FLBZ-CD_sc_ to rats improved its systemic exposure, resulting in significantly higher AUC_0-LOQ_ values compared to all other experimental groups (Table 1). Additionally, FLBZ was detected in plasma for longer period (up to 16 h post-treatment) after parenteral administration (FLBZ-CD_sc_ and FLBZ-TWEEN).

The administration of FLBZ as a Tween 80-based formulation to rats did not improve its systemic availability compared to oral administration of FLBZ-CD_oral_. Similar FLBZ AUC_0-LOQ_ values were observed in the FLBZ-CD_oral_ and FLBZ-TWEEN groups. In these groups, no differences were observed in either Cmax or AUC_0-LOQ_ values obtained for H-FLBZ (Table 1).

**Figure 4:**
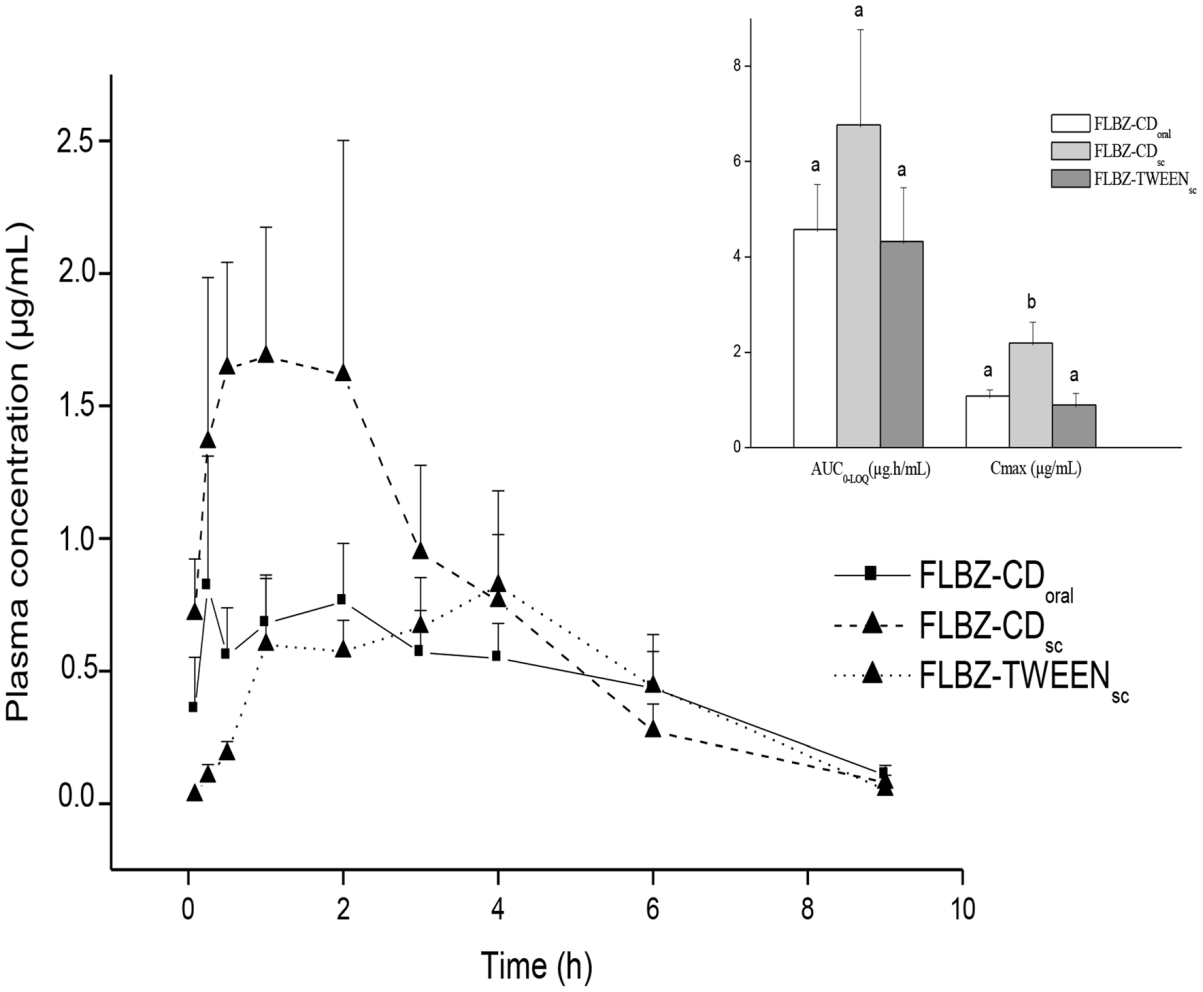
Flubendazole jird. Comparative flubendazole (FLBZ) mean (± SD) plasma concentration profiles obtained after administration of different formulations to uninfected jirds (n = 4 per time point). Insert shows the pharmacokinetic parameters AUC _0-t_ and Cmax (µg/mL) estimated for FLBZ parent drug. Different letters indicate statistically significant differences (P<0.05) between experimental groups.

**Figure 5:**
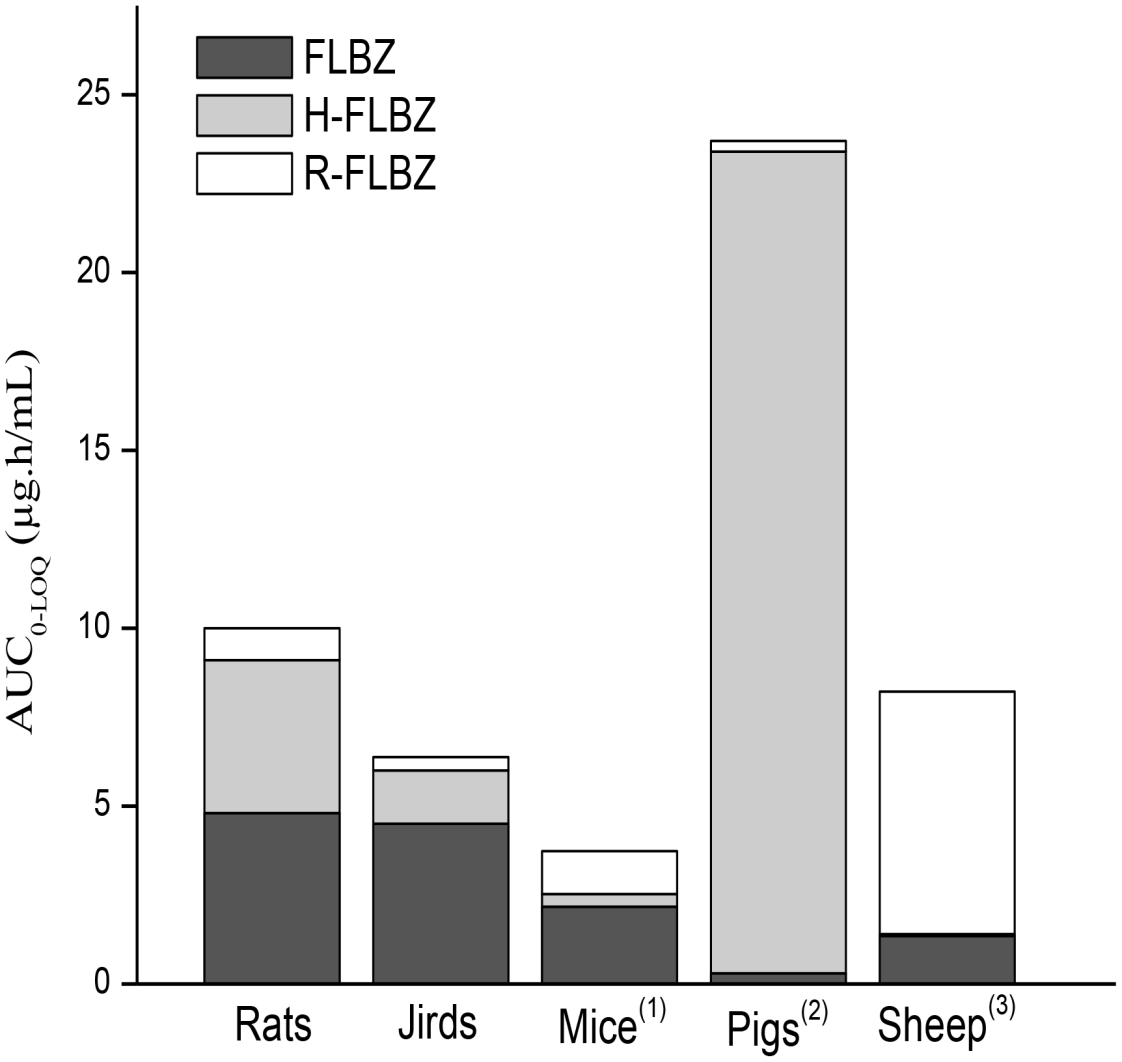
Relative systemic exposure (area under concentration vs. time curve, AUC _0-t_) of flubendazole (FLBZ) and its reduced (R-FLBZ) and hydrolyzed (H-FLBZ) metabolites in different animal species treated orally with a FLBZ-CD solution. Data taken from Ceballos et al, 2009 (1), 2012 (a) (2), 2012 (b) (3).

Unlike rats, neither H-FLBZ nor R-FLBZ was detected in plasma at any time post-treatment of jirds with FLBZ-CMC. Only trace amounts of FLBZ were detected, and only over a period so short that it precluded pharmacokinetic analysis. However, treatment with either FLBZ-CD (oral or sc routes) or FLBZ-TWEEN solutions allowed quantification of FLBZ and its reduced and hydrolyzed metabolites in jirds. Similar to rats, FLBZ was the main analyte detected in plasma, whereas H-FLBZ concentrations represented 10–20% of the total amount of drug recovered, with even lower R-FLBZ concentrations in jirds (Figure 2). The comparative plasma concentration profiles (mean ± SD) for FLBZ after administration as CD- or Tween 80-based formulations are shown in Figure 4. Table 2 summarizes the main pharmacokinetic parameters obtained for FLBZ after oral (FLBZ-CD_oral_) or sc (FLBZ-CD_sc_ and FLBZ-TWEEN) administration to jirds. The sc treatment with the Tween 80-based formulation did not improve FLBZ systemic exposure compared to FLBZ-CD_oral_; similar AUC_0-LOQ_ and Cmax values were obtained in both groups (Table 2). However, FLBZ absorption in the FLBZ-TWEEN group was slower compared to sc and oral FLBZ-CD groups, since a significantly longer Tmax was observed in that group. Interestingly, FLBZ-CD_sc_ delivered an enhanced FLBZ Cmax value (90–130%) compared to FLBZ-CD_oral_ and FLBZ-TWEEN. A similar trend was observed in AUC_0-LOQ_ values, but high individual variability may have obscured detection of statistically significant differences in this pharmacokinetic parameter among groups. Comparison of the relative contribution of FLBZ, H-FLBZ and R-FLBZ to the total drug plasma concentrations quantified after FLBZ treatment in different animal species, including rats, jirds, mice, pigs and sheep, is shown in Figure 5.

**Table 2:** Pharmacokinetic parameters (mean ± SD) obtained for flubendazole (FLBZ) after its administration (5 mg/kg) as different formulations to uninfected jirds: Cyclodextrin-based (FLBZ-CD_oral_ or FLBZ-CD_sc_) and Tween-based (FLBZ-TWEEN) formulations.

Pharmacokinetic parameters	FLBZ		
	FLBZ-CD_oral_	FLBZ-CD_sc_	FLBZ-TWEEN_sc_
**Cmax (µg/mL)**	1.08±0.17^a^	2.19±0.48^b^	0.89±0.29^a^
**Tmax (h)**	1.15±0.98^a^	0.82±0.78^a^	2.25±1.50^b^
**AUC_0-LOQ_ (µg.h/mL)**	4.57±0.99^a^	6.77±2.04^a^	4.32±1.17^a^

C_max_: peak plasma concentration; T_max_: time to peak plasma concentration; AUC_0**-LOQ**_: area under the concentration vs. time curve from 0 up to the limit of quantification. *Different letters indicate statistically significant differences (P<0.05) between experimental groups*.

## Discussion

When pharmacological research cannot be done on humans for practical and ethical reasons, animal models constitute a practical approach to understand the parasite-active drug-host relationship. In the current work, two different animal models (rat and jird) were used to approximate what might be expected in humans. The overall plasma pharmacokinetic behaviour of BZD anthelmintics in humans is similar to other monogastric species such as mice or rats, and greatly differs from what has been reported in ruminant species (sheep, cattle). In ruminants, the rumen acts as a drug reservoir, and slowing the digesta transit time results in improved systemic availability as a consequence of greater dissolution of drug particles in the acidic pH of the abomasum (the stomach) [22]. The briefer gut transit time in monogastric species allows a shorter time for dissolution of the drug suspension compared to ruminants, limiting gastrointestinal absorption of the drug. Thus, rats or jirds could be valid animal models to obtain kinetic data extrapolatable to humans, particularly when systemic exposure of BZD anthelmintics is evaluated after the administration of different formulations. Additionally, jirds have been extensively used as animal models in drug screening studies for potential antifilarial compounds [8], [11]. Thus, the basic pharmacokinetic data reported here could be linked to efficacy trials against filarial nematodes in the same animal model.

Three main factors play important roles in activity against nematodes: i. attaining sufficient drug concentrations at the site of target parasite location to be able to therapeutically affect receptors in parasites [23]; ii. drug lipophilicity [22]; and iii. physicochemical features of the tissue/fluids surrounding the parasite [24]. Drug concentration at the site of parasite location depends on the chemical properties of the drug and the pharmaceutical preparations in which the active compound is formulated. Therapeutic failures observed in parasite control in both human and veterinary medicine may be related to exposure of parasites to sub-therapeutic drug concentrations due to poor drug dissolution and/or insufficient systemic availability of the active ingredient. Obtaining adequate drug concentrations in the compartment in which the parasite resides is a key factor that determines efficacy against systemic parasites. The physicochemical features of the parasite environment play a pivotal role in determining drug access and accumulation. Some nematode parasites living in host tissues may be protected from the deleterious effect of an anthelmintic due to low diffusion of lipophilic compounds. Furthermore, the low water solubility of BZD anthelmintics seriously limits their absorption and systemic bioavailability. Clearly, the poor oral absorption of FLBZ after administration in the conventional suspension/tablet formulations is a serious disadvantage for the treatment of systemic infections such as filariasis. Low FLBZ bioavailability has been associated with low *in vivo* activity against cystic echinococcosis in mice [17]. The use of pharmacotechnical strategies to overcome this limitation may markedly improve the *in vivo* efficacy of FLBZ against systemic parasitic nematodes.

The lack of water solubility is an important limitation for the formulation of the most potent BZD methylcarbamate anthelmintics, such as FLBZ. Irritation and post-injection precipitation are concerns in parenteral drug delivery for poorly water-soluble drugs [25]. The greater water solubility of the main active albendazole metabolite, albendazole sulphoxide (also named ricobendazole), was the starting point in the development of an injectable formulation for use in cattle currently available in some Latin American countries [26]. However, since BZD aqueous solubility is markedly higher at low pH values [27], that formulation contains ricobendazole (15% final concentration) at low pH (approx. 1–2), which produces irritation at the site of sc administration. Recently, a CD-based formulation of ricobendazole for parenteral use has demonstrated adequate tissue tolerability and bioavailability [25], but is not available in the veterinary market. Complexation with cyclodextrins has been intensively investigated as a solubilization approach for parenteral formulations. In agreement with our recent results, cyclodextrin formulations of poorly water-soluble drugs have shown little or no tendency for drug precipitation after intramuscular injection [25]. It is worth noting that most of the progress achieved to improve bioavailability of BZDs has been in formulation design [18], [28]–[29].

Prospects for an accelerated path to the elimination of onchocerciasis and lymphatic filariasis would be much enhanced if a safe and effective macrofilaricide were available [5], [6], [7]. Therefore, improvement of FLBZ systemic availability was the essential component under evaluation in the current work. We have previously reported that CDs markedly increase FLBZ water solubility [16], which was correlated with enhanced systemic drug exposure in mice [17], as demonstrated by significantly higher plasma Cmax (28 fold-higher) and AUC (27 fold-higher) values compared to a conventional suspension. Moreover, the efficacy of FLBZ against cystic echinococcosis in mice was also dramatically improved after oral administration of a CD-based formulation [17], [30]. Similar pharmacokinetic results were obtained for ABZ in mice [18], [30] and humans [19], in which a significantly higher systemic exposure for ABZ-sulphoxide was observed after ABZ administration in a CD-based solution. Consistent with previous data, the CD-based formulations FLBZ-CD_oral_ and FLBZ-CD_sc_ significantly increased FLBZ systemic exposure in rats compared to the FLBZ-CMC formulation. Similar FLBZ AUC_0-LOQ_ values were observed between the FLBZ-CD_oral_ and the Tween 80-based sc formulations. The highest FLBZ relative plasma availability was attained in the FLBZ-CD_sc_ group, in which the AUC_0-LOQ_ value increased by 684% compared to that observed after the oral administration of the FLBZ-CMC suspension. The FLBZ plasma detection period (up to 16 h post-treatment) was similar among the CD- and Tween 80-based formulations. CDs have the ability to complex with drugs, affording increased water solubility and improved oral bioavailability of FDA Class II compounds (poor aqueous solubility, high permeability) [15], such as the BZD anthelmintics. In the current work, the CD-based formulation induced drastic changes in FLBZ aqueous solubility, which accounted for its enhanced absorption and systemic availability in rats.

Interestingly, neither FLBZ nor its metabolites were detected in plasma after FLBZ-CMC treatment in jirds. Similar FLBZ plasma AUC_0-LOQ_ values were observed among FLBZ-CD oral or sc treatments and the FLBZ-TWEEN groups. The H-FLBZ and R-FLBZ metabolites were recovered in plasma, although in much lower concentrations than the parent drug. In agreement with kinetic data obtained in rats, high FLBZ peak plasma concentrations were observed after sc administration as a CD-solution compared to the Tween 80 sc formulation. As previously mentioned, CD clearly improves FLBZ absorption in jirds after both oral or sc treatment. It is generally accepted that CDs enhance drug permeability by solubilizing their lipophilic components, thereby disrupting barriers to diffusion and increasing permeability. CDs may also act as permeation enhancers by carrying the drug in inclusion complexes through the aqueous barrier, from the bulk solution towards the surface of biological membranes [31].

Dominguez-Vazquez et al. [12] demonstrated that an injectable formulation of FLBZ was highly efficacious in humans against adult *O. volvulus*. Parenteral administration of a FLBZ Tween 80-based formulation has high efficacy against multiple filarial species in several animal hosts [6]–[8]. Since FLBZ plasma exposure obtained in the FLBZ-CD_oral_ and FLBZ-CD_sc_ groups was greater than obtained in the FLBZ-TWEEN group, high macrofilaricidal efficacy of the CD-based formulations may be possible. However, the potential of those formulations for treatment of humans may be limited by the high cost of the CD used in the formulation assessed in the current experimental work (hydroxyl propyl β-cyclodextrin).

Unlike other commonly used BZD anthelmintics, such as albendazole (aliphatic substitution at position -5) and fenbendazole (aromatic substitution at position -5), FLBZ contains a ketone group in that position, which has implications for its metabolism by the host. While sulphur-containing BZDs are sequentially oxidised to their sulphoxide and sulphone metabolites by both flavin-monooxygenase (FMO) and cytochrome P450 (P450) systems in the liver [32]–[33], carbonyl reductases (CBRs) are thought to be the main enzymes involved in FLBZ biotransformation [34]. The main FLBZ metabolic pathways include reduction of the ketone group to form R-FLBZ, and hydrolysis of the methylcarbamate group to form H-FLBZ. The contribution of each metabolite to the total amount of drug recovered from plasma after FLBZ treatment may vary among animal species. R-FLBZ is the main metabolite measured in plasma after FLBZ treatment in sheep [10], [16] and mice [17], [30] (Figure 5). However, while low plasma concentrations of FLBZ are detected in sheep, the parent compound was the main analyte in FLBZ treated mice. A different pattern was observed in pigs treated with FLBZ, in which H-FLBZ was the predominant molecule, representing 97% of total drug measured in the bloodstream after FLBZ treatment [35] (Figure 5). In rats, similar amounts of FLBZ and H-FLBZ were present in the bloodstream, with only trace amounts of the R-FLBZ metabolite. Although oral bioavailability of FLBZ has been estimated in humans [9], no data are available on the plasma pharmacokinetic pattern of FLBZ and metabolites. However, *in vitro* studies performed in our lab have shown that human microsomes biotransform FLBZ mainly to the R-FLBZ metabolite (unpublished data), which suggests similarity with the metabolic profile observed in mice and sheep (see Figure 4). Species-related differences in plasma drug exposure observed for FLBZ and metabolites may significantly influence drug efficacy. While H-FLBZ is an inactive metabolite, biological activity has been described for R-FLBZ [30], [36]–[37], which may contribute to anthelmintic efficacy observed after FLBZ treatment.

The marked improvement of FLBZ systemic availability observed after the administration of CD-based formulations to rats and jirds needs to be considered in terms of its potential usefulness as a macrofilaricide in animal models. If oral and/or parenteral administration of FLBZ-CD formulations provides satisfactory efficacy, the empirical correlation of plasma concentrations and efficacy may contribute to the development of new formulations for use in humans. The work reported here indicates that FLBZ plasma availability can be markedly improved by changes in formulation. The enhanced systemic exposure observed after treatment with the CD-based formulations has significant therapeutic implications for a drug with poor or erratic bioavailability.
